# Co-expression and interaction network analysis reveals dysregulated neutrophil and T-cell activation as the core mechanism associated with septic shock

**DOI:** 10.3389/fgene.2023.1132361

**Published:** 2023-02-23

**Authors:** Shaobo Zhao, Kun Zhu, Xiaoyi Li, Xiaohui Zhong, Yanan Zhao, Zhenkai Le, Zhicong Liu, Yi Xiao, Dengming Lai, Na Jiao, Qiang Shu

**Affiliations:** ^1^ Department of Pediatric Surgery, The Children’s Hospital, Zhejiang University School of Medicine, Hangzhou, Zhejiang, China; ^2^ Department of Pathology, The Children’s Hospital, Zhejiang University School of Medicine, Hangzhou, Zhejiang, China; ^3^ National Clinical Research Center for Child Health, The Children’s Hospital, Zhejiang University School of Medicine, Hangzhou, Zhejiang, China

**Keywords:** bioinformatics, neutrophil, ELANE, T cell, septic shock

## Abstract

Septic shock as a subset of sepsis, has a much higher mortality, while the mechanism is still elusive. This study was aimed at identifying core mechanisms associated with septic shock and its high mortality by investigating transcriptome data. We screened 72 septic-shock-associated genes (SSAGs) with differential expression between septic shock and sepsis in the discovery dataset. Further gene set enrichment analysis identified upregulated neutrophil activation and impaired T-cell activation in septic shock. Co-expression analysis revealed nine co-expressed gene modules. In addition, we determined twenty-one prognostic SSAGs using cox regression analysis in an independent dataset. Moreover, protein–protein interaction (PPI) network revealed two clusters. Among these neutrophil activation was enriched in the most positively-related modules and the cluster2 PPI network, while T-cell activation was enriched in both the most negatively-related module and one of the most positively-related modules as well as the cluster1 PPI network. ELANE, LCN2 and IFI44 were identified as hub genes with CytoHubba methods and semantic similarity analysis. Notably, ELANE was the only prognostic gene and was further validated in an external dataset. Blood neutrophil count was demonstrated to increase in septic shock and be a risky factor of prognosis based on clinical data. In conclusions, septic shock is associated with upregulated neutrophil activation and dysregulated T-cell activation. Three hub genes might have potentials as sensitive markers for the further translational research and ELANE could be a robust prognostic biomarker and effective therapeutic target.

## 1 Introduction

Sepsis is known as life-threatening organ dysfunction due to a dysregulated host response to infection (Singer et al., 2016), which has been a major global health concern because of high mortality and unacceptable hospital costs (Reinhart et al., 2017). A recent study reported a total of 11.0 million sepsis-related deaths in an estimated 48.9 million incident cases of sepsis worldwide in 2017 (Rudd et al., 2020). More importantly, the incidence of sepsis has still steadily increased over the past several decades (Esposito et al., 2017). Meanwhile, sepsis has been the most expensive condition for hospital stays in the United States, and the costs continue to increase (Liang et al., 2006; Torio and Andrews, 2006; Torio and Moore, 2006). In particular, septic shock, as a subset of sepsis with underlying circulatory and cellular/metabolic abnormalities (Singer et al., 2016), has a much higher mortality approaching 40%–60% than 10% of sepsis (Cecconi et al., 2018; Napolitano, 2018).

However, the significant biological and clinical heterogeneity of sepsis remains a major challenge, which has led to the failure of clinical sepsis trials of immunotherapy (Rubio et al., 2019). The understanding of sepsis and septic shock is still limited and keeps evolving over time. The Third International Consensus Definitions for Sepsis and Septic Shock (Sepsis-3) was developed in 2016 (Singer et al., 2016), reflecting improved knowledge on the pathophysiology of sepsis and septic shock. Importantly, Sepsis-3 led to the new definition of septic shock by a more restrictive and unambiguous criteria that the criteria of sepsis and vasopressor therapy needed to elevate mean arterial pressure ≥65 mmHg and lactate >2 mmol/L (18 mg/dL) despite adequate fluid resuscitation (Shankar-Hari et al., 2016; Singer et al., 2016; Napolitano, 2018), which means distinguishing septic shock from sepsis more clearly than ever before. In this context, it could be necessary to determine core mechanisms under the new definition for a more accurate interpretation of septic shock.

Previous studies of septic shock based on different definitions have shown several important mechanisms. Tissue hypoxia has been discussed as an important pathophysiological mechanism under the action of microbial endotoxins during septic shock (Pavez et al., 2020). From an immunological perspective, the activation of monocytes, macrophages and neutrophils was considered to participate in the intimate mechanism of septic shock (Gorecki et al., 2021). In particular, polymorphonuclear neutrophils (PMNs) have been shown to lose their direct antimicrobial functions and acquire an immunosuppressive action and participate in the generation of disseminated intravascular coagulation (DIC) when septic shock develops (Stiel et al., 2018). However, few studies have focused on the difference between septic shock and sepsis without shock syndromes. The mechanism of septic shock is not yet fully understood, and the identification of the core mechanism is still needed.

In this study, we analyzed the gene expression profiles of patients between septic shock and sepsis from public databases to identify core mechanisms associated with septic shock and its high mortality. Weighted gene co-expression network analysis (WGCNA) was conducted to identify septic-shock-associated gene modules. Prognostic genes among septic-shock-associated genes (SSAGs) were identified to explain the higher mortality at the molecular level. Combining the protein–protein interaction (PPI) network and semantic similarity network based on gene annotation, hub genes were identified with the most connectivity among SSAGs. The main goal of the present study was to better understand the molecular changes and screen core mechanisms responsible for the development from sepsis to septic shock under the new Sepsis-3 definition. For more accurate interpretation, the “sepsis” declared after in this study refers specifically to sepsis without shock diagnosis.

## 2 Materials and methods

### 2.1 Data source

The included transcriptome data were downloaded from gene expression omnibus (GEO) databases (http://www.ncbi.nlm.nih.gov/geo/) (Barrett et al., 2013). Only peripheral blood samples collected within 24 h of diagnosis or ICU admission were included. The RNA sequencing data of 91 adult samples (including 19 septic shock, 20 sepsis, 12 uncomplicated infection and 40 healthy controls) in the GSE154918 dataset, which were pre-processed using the DESeq2 package by the contributors (Love et al., 2014; Herwanto et al., 2021), were used as discovery dataset to explore genes, modules and mechanisms associated with septic shock. Additionally, the array data and survival information of 479 adult sepsis samples with a 28-day cumulative death rate about 23.80% in the GSE65682 dataset were read in R language to determine the prognostic significance of interested genes in sepsis patients. The gene expression profiles of GSE65682 were background-subtracted and normalized by a robust multi-array average algorithm using the affy package. The row count matrix of 345 adult sepsis samples including 52 dead and 293 survival samples in the GSE185263 dataset was downloaded to validate survival significance of the hub gene.

Clinical blood laboratory examinations data of sepsis and septic shock patients were extracted from the MIMIC-IV (version 2.0) database in the physionet (https://physionet.org/content/mimiciv/2.0/) for the further validation (Goldberger et al., 2000; Johnson et al., 2022). One of the authors who has finished the required Collaborative Institutional Training Initiative examination (Certification number 53459610 for Zhao) can access the database. The adult ICU stay samples meeting the sepsis-3 definition at the first day of ICU admission were included (Singer et al., 2016). The patients’ parameters including absolute neutrophil count, absolute CD3 count (i.e., T cell count), absolute CD4 count and absolute CD8 count from blood specimens and survival data were extracted for further analysis. Specifically, we extracted the max values of neutrophil counts of each ICU stay within 6 h before ICU admission and 24 h after; while the chart time requirements of the other three items were limited to 6 h before ICU admission and 48 h after, concerning their more time costs waiting for the reports. In our study, the data about neutrophil counts of 8250 ICU stays containing 40.5% septic shock samples and with a 28-day cumulative mortality rate (CMR) about 22.3% were extracted. However, among them only 69 had the time-limited data about CD3 counts and 68 had desirable CD4 counts and CD8 counts due to their less clinical applications. More details were shown in Supplementary Table S1. The code used for data extraction can be available on GitHub (https://github.com/MIT-LCP/mimic-iv).

### 2.2 Differential gene expression analysis

Differential expression analysis was conducted using moderated *t*-test by the limma R package (Ritchie et al., 2015). The differential expression cutoff values were set to |log2 fold change (logFC)|≥ 1 and adjusted *p*-value (adj.P) < 0.05. *p* values were adjusted by the Benjamini–Hochberg (BH) method.

### 2.3 Functional enrichment analysis

Functional enrichment analysis was conducted using the clusterProfiler R package (Yu et al., 2012). Gene set enrichment analysis (GSEA) based on the rankings of logFC of all genes and over-representation analysis was utilized to determine enriched biological process (BP) GO terms and KEGG pathways. The cutoff of the adjusted *p*-value by the BH method was set to 0.05.

### 2.4 Weighted gene co-expression network analysis (WGCNA)

The weighted co-expression network was constructed using the WGCNA package (Zhang and Horvath, 2005; Langfelder and Horvath, 2008). The minimum module size was set to 30, the dendrogram cut height for module merging was set to 0.2 and the desired minimum scale free topology fitting index *R* ^*2*^ was set to 0.8 to screen optimal soft-thresholding power. Module eigengene (ME) was defined as the first principal component of the gene expression matrix of the corresponding module. The relationships between model eigengenes and phenotypes were assessed using the Spearman correlation.

### 2.5 Survival analysis

A univariate Cox proportional hazard regression model was conducted by the survival R package to screen prognostic factors from septic-shock-associated DEGs in an independent dataset (GSE65682), and *p* values were corrected by the BH method.

### 2.6 Protein-protein interaction (PPI) network analysis

The PPI network were constructed based on the DEGs of septic shock online in the STRING database (http://string-db.org/) (version 11.5) (Szklarczyk et al., 2021). Specifically, the gene list was input into the multiple protein mode with default parameters. The credibility was set to 0.40. Disconnected nodes were hidden. Then, the output table was input into Cytoscape software. CytoHubba, a Cytoscape plugin, was used to screen potential hub genes by providing 12 topological analysis algorithms (i.e., MCC, DMNC, MNC, Degree, EPC, Bottleneck, Eccentricity, Closeness, Radiality, Betweenness, Stress, and Clustering Coefficient) (Chin et al., 2014). In this research, genes appearing at least 5 times in the top 10 results of each algorithm were considered as potential hub genes.

### 2.7 Semantic similarity analysis

Semantic similarities were measured using the GOSemSim package (Yu et al., 2010; Yu, 2020). The pairwise semantic similarities were calculated by Wang’s measure algorithm (Wang et al., 2007) from three aspects, including biological processes (BP), molecular function (MF) and cellular component (CC). The final adjacency matrix of semantic similarities between genes was identified as the geometric means of the similarities from these three aspects. The candidate hub genes were screened according to the decreasing order of average semantic similarities of each gene.

### 2.8 Immune cell correlation estimation

To estimate the immune cell fractions, CIBERSORTx, a suite of machine learning tools (https://cibersortx.stanford.edu/), was used to perform a deconvolution algorithm based on bulk expression profiles (Newman et al., 2019). The correlation between hub genes and cell fractions in the GSE65682 dataset was estimated using the Spearman rank correlation coefficient.

### 2.9 Clinical investigation of neutrophil and T-cell counts

To further validate the association**s** of neutrophils and T-cells with septic shock, the difference**s** of the neutrophil counts, CD3 counts, CD4 counts and CD8 counts between septic shock and sepsis were accessed using two-sample Wilcoxon rank sum test, where the criteria of the statistical significance was set to *p* < 0.05 (two-sided). The prognostic association**s** were accessed using the univariate Cox proportional hazard regression model by the survival R package**.** Kaplan-Meier (KM) curves were further performed by the survminer R package to evaluate prognostic association in different subgroups of sepsis. The optimized cut-off values of each group were identified using X-tile tool respectively (Camp et al., 2004).

### 2.10 Software and version

R (version x64 3.6.2) and Cytoscape (version 3.8.2) were used through the analysis. The artworks were created by Adobe Illustrator CC (version 64-bit 22.1).

## 3 Results

### 3.1 Identification of differentially expressed genes

**FIGURE 1:**
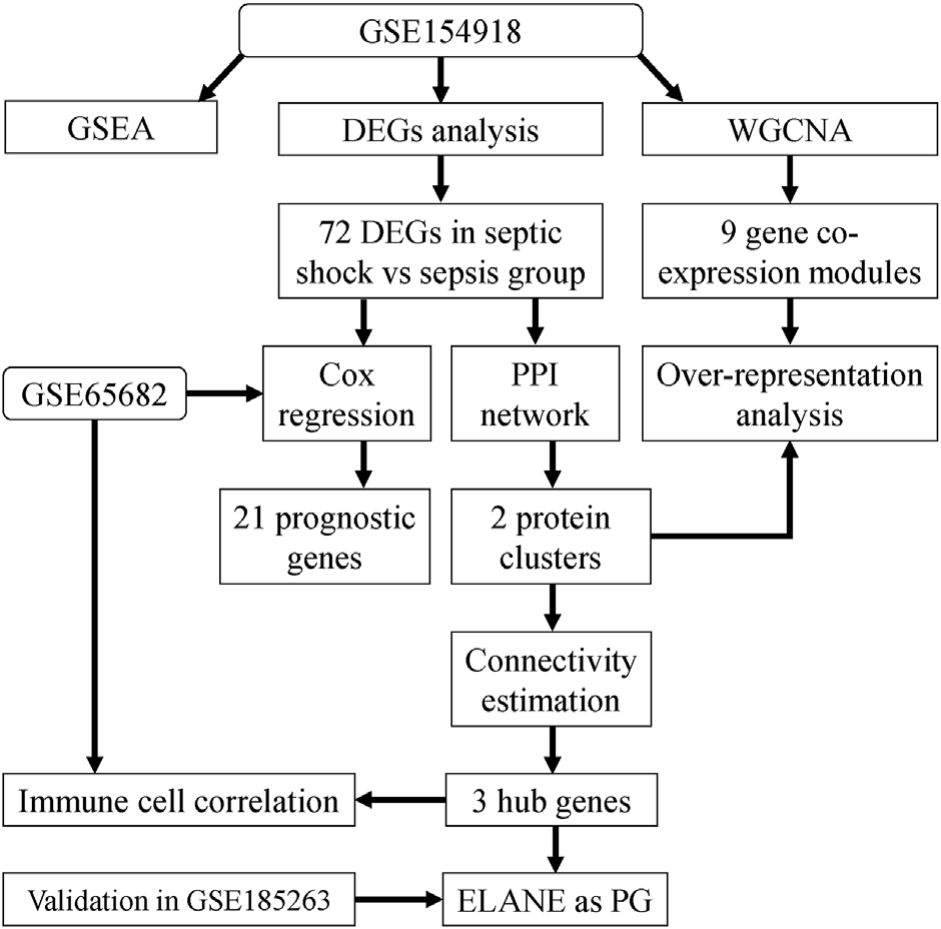
Study workflow. Bioinformatics analysis to screen core molecular mechanisms associated with septic shock and its high mortality. PPI, protein-protein interaction networks; GSEA, gene set enrichment analysis; WGCNA, weighted gene co-expression network analysis; DEG, Differential expressed gene; PG, prognostic gene.

**FIGURE 2:**
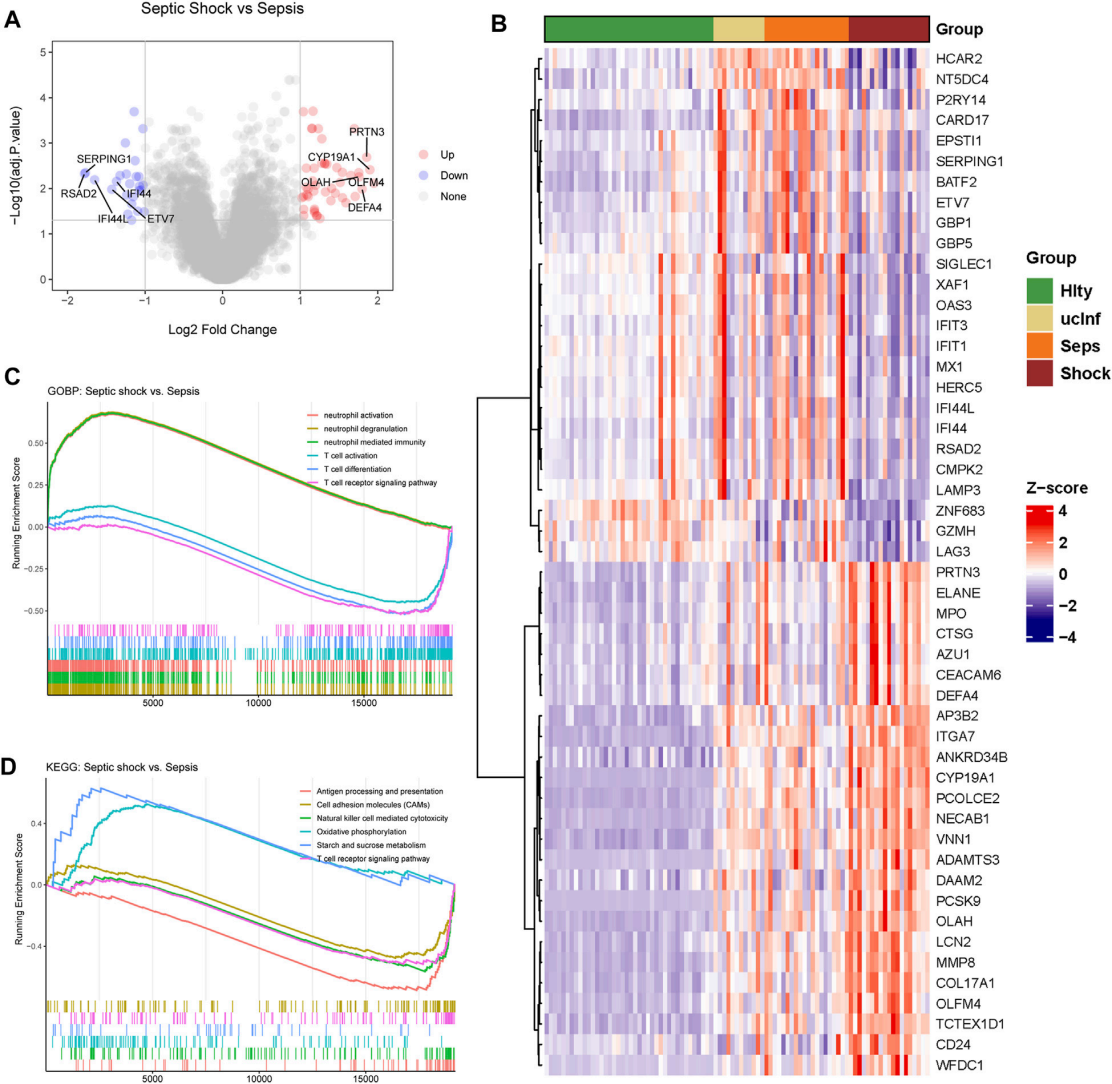
Differential gene expression between septic shock and sepsis patients. **(A)** Differently expressed genes in septic shock (n = 19) vs. sepsis (n = 20) group. **(B)** The expression heatmap of 25 top upregulated DEGs and 25 top downregulated DEGs. Hlty, healthy control; ucInf, uncomplicated infection; Seps, sepsis; Shock, septic shock. **(C)** Biological processes enriched in septic shock vs sepsis groups. **(D)** KEGG pathways enriched in septic shock vs. sepsis groups.

As shown in the flowchart in Figure 1, we first analyzed genes differentially expressed between septic shock (n = 19) and sepsis (n = 20) groups in the discovery (GSE154918) dataset. A total of 72 septic-shock-associated DEGs were identified as septic-shock-associated genes (SSAGs) with |logFC>1| and adj.*p* < 0.05 (Figure 2A), among which 47 genes were upregulated and 25 genes were downregulated in septic shock. The heatmap showed the overall trend among the course from healthy to septic shock of the top 25 upregulated and 25 downregulated genes (Figure 2B). Of note, most of the DEGs showed significant increases from healthy to infection group, reflecting their possible participation in the infection-driven mechanisms.

### 3.2 Septic shock showed excessive neutrophil activation and impaired T Cell activation

To explore the mechanism of the development of septic shock, gene set enrichment analysis (GSEA) was used to provide global insight to assess the gene expression patterns of septic shock (Figures 2C, D).

Compared with sepsis group, we found that in septic shock, significant upregulation of the biological processed related to neutrophil activation, and those related to T cell activation were significantly downregulated (Figure 2C). Pathways related to energy metabolism were significantly upregulated, while the pathways including antigen presentation, T cell receptor (TCR) signaling pathways and NK cell mediated cytotoxity were downregulated (Figure 2D). Excessive neutrophil activation and impaired T cell activation could be the major characteristics of septic shock.

### 3.3 Identification of septic-shock-associated co-expression gene module

**FIGURE 3:**
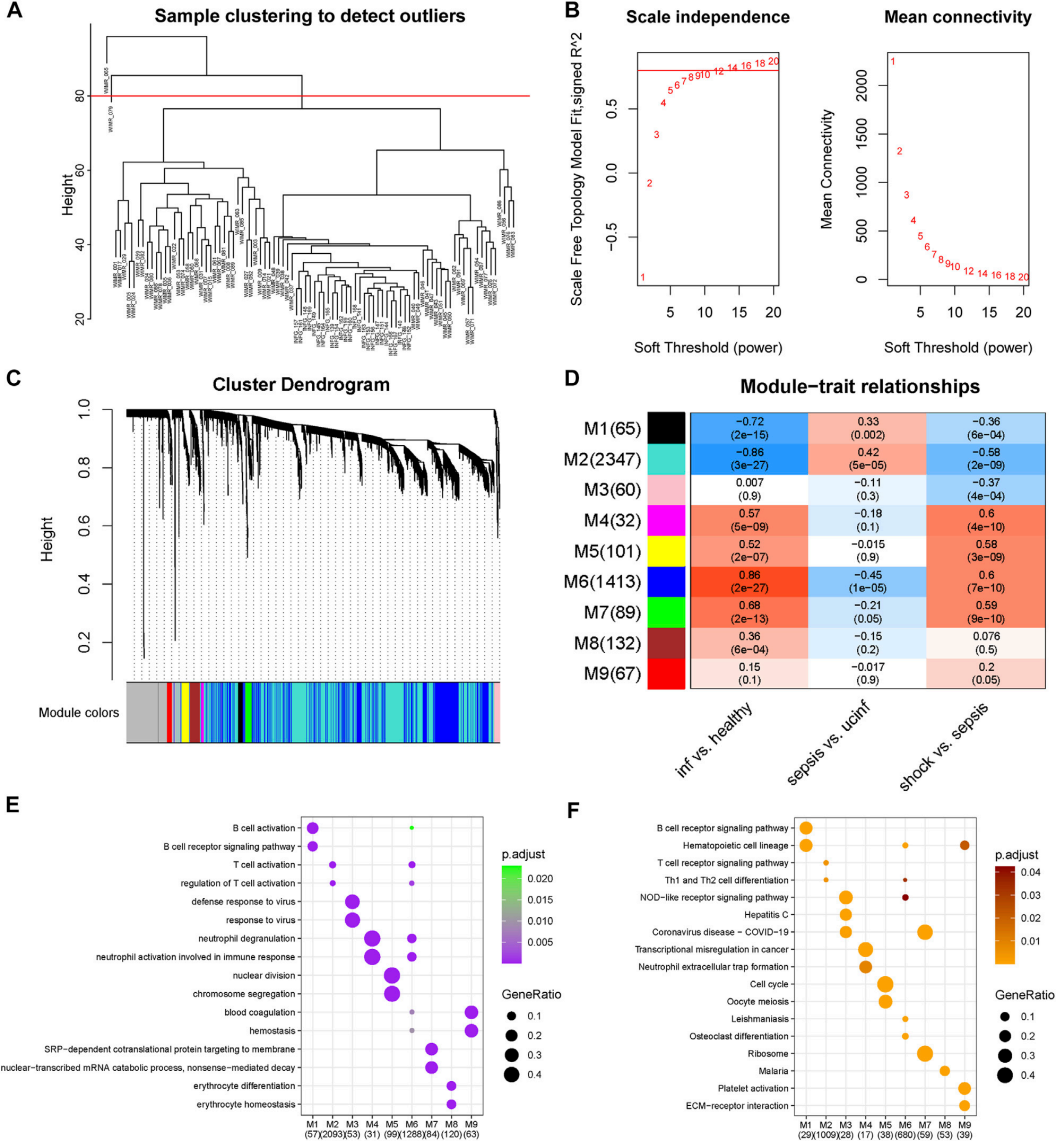
Co-expression network construction. **(A)** Hierarchical clustering dendrogram of adopted samples to detect outliers. **(B)** Determination of optimal soft-thresholding power. **(C)** Hierarchical clustering dendrogram and corresponding modules of involved genes. **(D)** Correlation heatmap of module eigengenes and phenotypes. The color of the squares gradually from blue to red represents the Spearman correlation coefficients. **(E)** Biological processes enriched in each module, the color and the size of the dots corresponded to the significance of enrichment and the ratio of enriched gene numbers in the corresponding terms respectively. **(F)** KEGG pathways enriched in each module, the color and the size of the dots corresponded to the significance of enrichment and the ratio of enriched gene numbers in the corresponding terms respectively.

After excluding two outliers and setting soft-thresholding power to 14 (Figures 3A, B), a total of nine co-expression modules were identified based on the expression profiles of 5,000 genes with most median absolute deviation (Figure 3C). Correlational analysis between modules and phenotypes revealed positive correlations with septic shock of M4, M5, M6 and M7 and negative correlations of M1, M2 and M3. Moreover, M4 and M6 were shown to be the most positively related module to septic shock, while M2 showed the most negative correlation (Figure 3D).

Further over-representation analysis showed M4 and M6 enriched in processes and pathways about neutrophil activation while M2 and M6 enriched in those about T cell activation, suggesting the activation of neutrophil and T cell activation as key mechanism of septic shock (Figures 3E, F). In addition, M5 and M7, which showed nearly the highest positive correlations, were enriched in cell-division-related processes and RNA-metabolism-related processes, respectively.

Of note, we found modules correlated with neutrophil and T cell activation in part showed different trend among the step course from healthy to septic shock (Figure 3D), especially M2 related to T cell activation showed the most negative correlation with septic shock and infection as well as the most positive correlation with sepsis, meanwhile M6 both related to neutrophil and T cell activation as one of the most positively correlated modules with septic shock and infection showed the most negative correlation with sepsis. M4 module, which was related to neutrophil activation especially neutrophil extracellular trap (NET) formation, showed positive correlation with infection and septic shock while unsignificant correlation with sepsis. These findings further validate the specific transcriptomic changes from sepsis to septic shock.

However, T-cell-related modules were observed more perplexing associations that M6 was positively related and M2 was negatively related to septic shock, meanwhile M6 was also related to neutrophil activation. To further understand the functions of M6 in T cell activation, the gene-concept networks were constructed based on the enrichment analysis of M6 genes and their gene significances for septic shock (Supplementary Figures S1, S2). We found neutrophil-related genes showing highly consistent up-regulations while T-cell-related genes showed correlations in different directions and no obvious distribution tendency was observed. Interestingly, several HLA (Human leukocyte antigen) Class-II molecules, specifically HLA-DPA1 (Major Histocompatibility Complex, Class II, DP Alpha 1), HLA-DQA1 (Major Histocompatibility Complex, Class II, DQ Alpha 1), HLA-DRB1 (Major Histocompatibility Complex, Class II, DR Beta 1), HLA-DMB1 (Major Histocompatibility Complex, Class II, DM Beta 1) and HLA-DPB1 (Major Histocompatibility Complex, Class II, DP Beta 1), were all downregulated. On the other hand, the downregulated TCR signalling pathway were inferred based on the results of GSEA and the enrichment analysis for M2 genes (Figures 2C, D; Figure 3F). Combining these two findings, the suppression of the interaction of TCR and HLA-II might be an important part of the mechanism. Beyond that, the mechanism of dysregulation of T cell activation was still seemed more complicated.

### 3.4 Identification of septic-shock-associated prognostic genes

**FIGURE 4:**
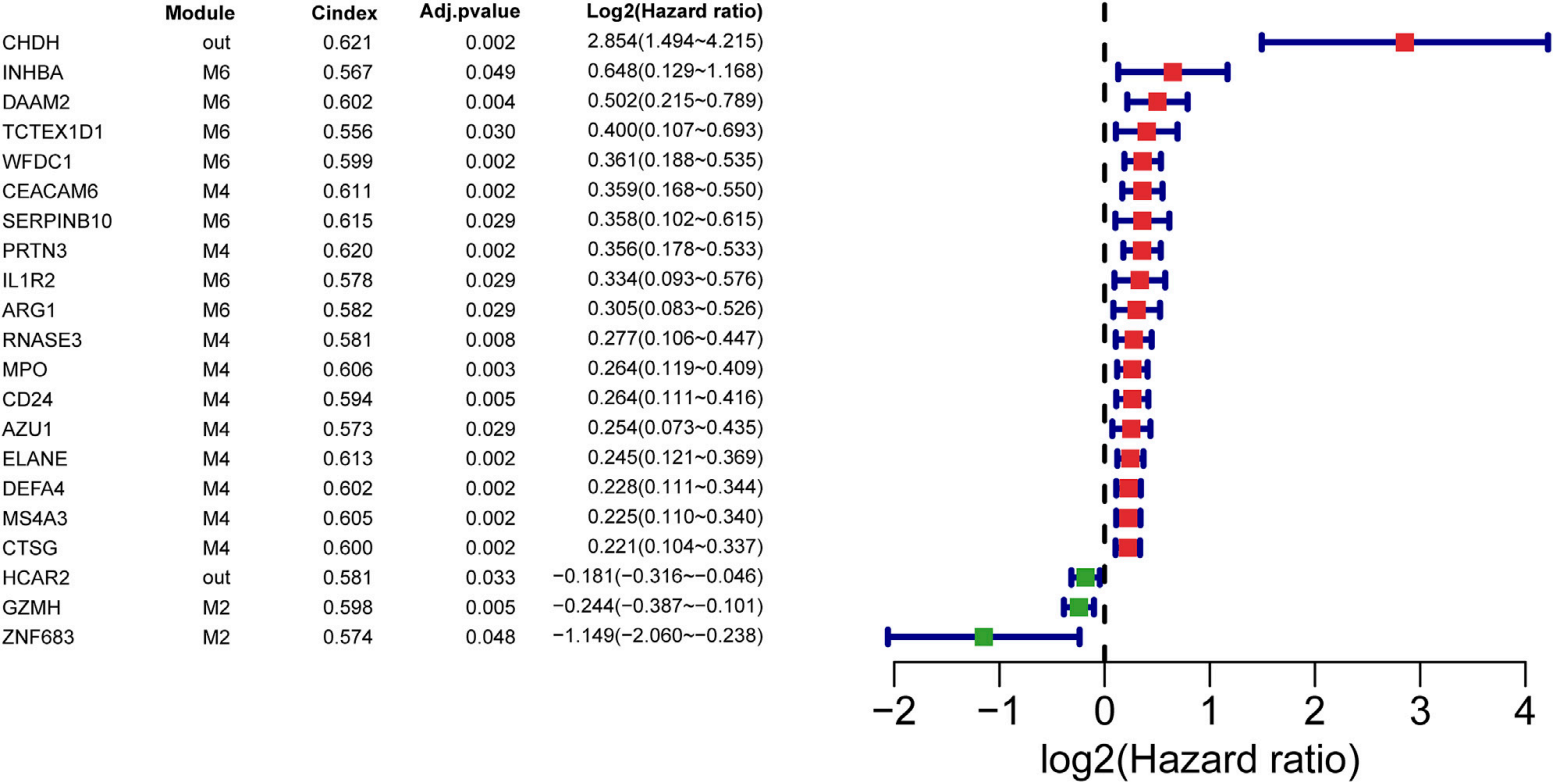
Prognostic SSAGs. The forest plots of prognostic SSAGs with adjusted *p*-value less than 0.05 by univariate cox analysis in the GSE65682 dataset. Red forest plots represent risky factors and green forest plots represent protective factors.

To identify important genes associated with high mortality of septic shock. Septic-shock-associated DEGs with adjusted *p*-value <0.05 using univariate Cox analysis were further screened as septic-shock-associated prognostic genes. As a result, 21 genes were screened with significant correlations with 28-day cumulative death (Figure 4). Among them, 18 genes were identified as risky factors (log2HR > 0) and three genes were identified as protective factors. Notably, most of the risky factors belonged to M2, M4 and M6, which were identified as modules mainly related to neutrophil and T cell activation. Moreover, M2 showed the most negative correlation, and M4/M6 were two of the most positively correlated modules, reflecting their major association with septic shock and its high mortality.

### 3.5 Construction of PPI network

**FIGURE 5:**
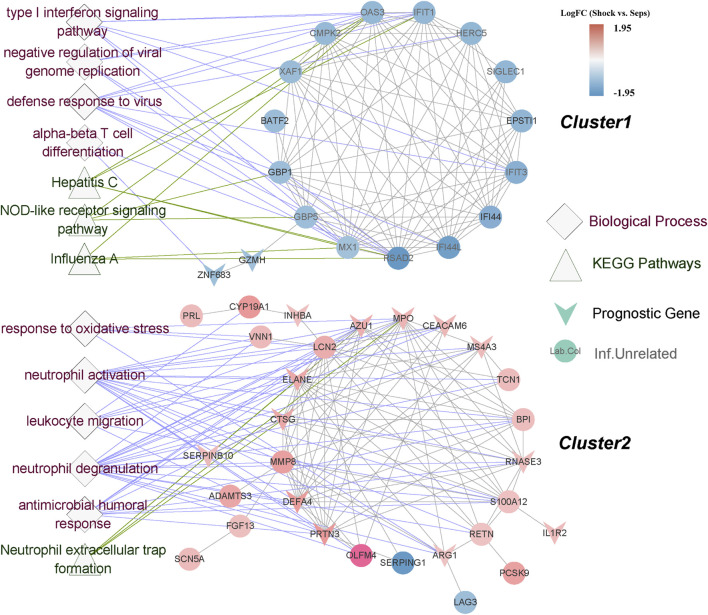
PPI network construction and over-representation analysis of cluster genes. PPI network constructed by SSAGs from STRING database, were clearly divided into 2 clusters. The color of gene nodes indicated log2 fold change. V-shape represented the prognostic genes. The diamond shaped nodes and the triangle shaped nodes represented enriched biological processes and KEGG pathways correspondingly.

The PPI network based on 72 DEGs was distinctly separated into two clusters, and each of them showed high consistency of the trend of expression differences (Figure 5). All of the cluster1 genes were downregulated in septic shock, while almost all of the cluster2 genes were upregulated. Interestingly, most of the prognostic genes were concentrated in cluster2. We also found that most cluster1 genes did not show significant difference compared with healthy group. Functional over-representation analysis revealed the significant enrichment of response to virus and NOD-like receptor signaling pathway for the cluster1 genes, while neutrophil-related processes and pathways such as neutrophil degranulation process and neutrophil extracellular trap (NET) formation pathway enriched in the cluster2 genes.

### 3.6 Identification of septic-shock-associated hub genes

**FIGURE 6:**
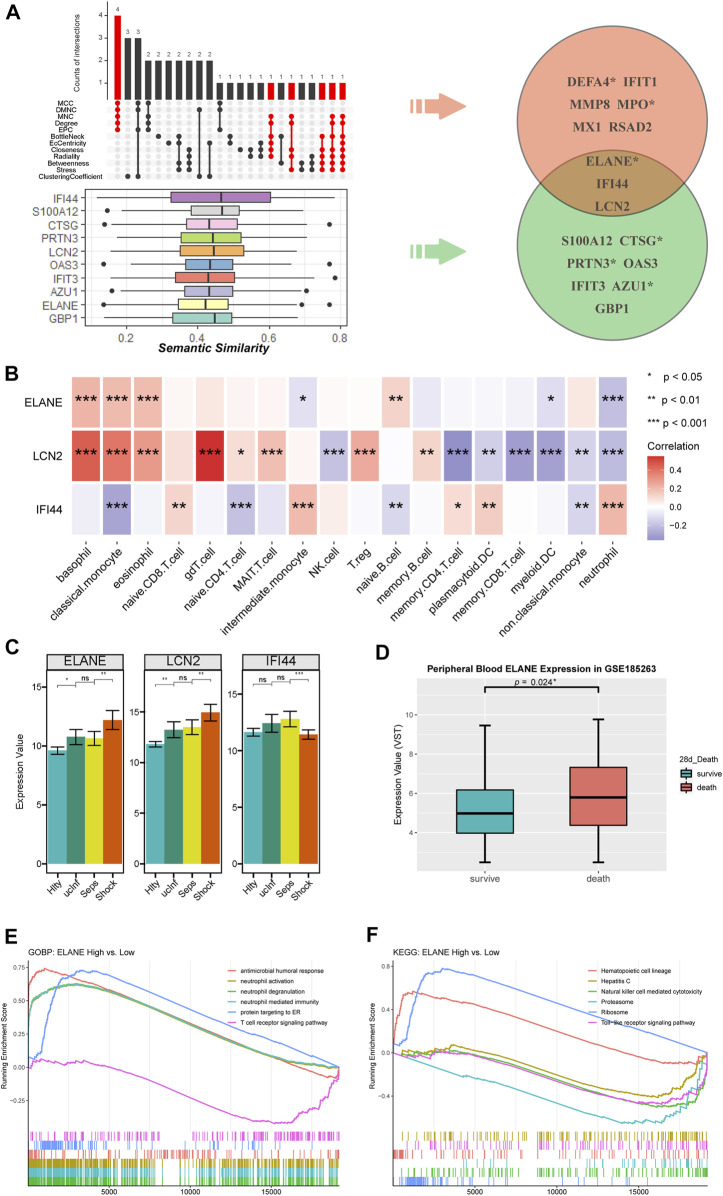
The identification of hub genes in SSAGs. **(A)** The genes appearing at least 5 times in top 10 results of each algorithm using cytoHubba were extracted (marked in red) and shown in the upset plot, below the SSAGs with top 10 functional similarities estimated by Semantic similarity analysis were shown in box plots. Three genes were identified as hub genes by overlapping the two results. The gene names marked with an asterisk indicated them as prognostic genes. **(B)** Correlations between hub genes and immune cell fractions. **(C)** Expression values of hub genes among the course from healthy to septic shock. **(D)** ELANE was significantly upregulated in death (n = 52) vs. survival (n = 293) group of the GSE185263 dataset. **(E)** Biological processes enriched in ELANE-high group. **(F)** KEGG pathways enriched in ELANE-high group.

Combining the results of 12 algorithms of cytoHubba app, a total of nine hub genes, including DEFA4 (Defensin Alpha 4), IFIT1 (Interferon Induced Protein With Tetratricopeptide Repeats 1), MMP8 (Matrix Metallopeptidase 8), MPO (Myeloperoxidase), MX1 (MX Dynamin Like GTPase 1), RSAD2 (Radical S-Adenosyl Methionine Domain Containing 2), ELANE (Elastase, Neutrophil Expressed), IFI44 (Interferon Induced Protein 44) and LCN2 (Lipocalin 2), were identified with at least five appearances in the top 10 results of each algorithm (Figure 6A).

On the other hand, based on the functional similarity among SSAGs calculated by the GOSemSim method, the hub genes including ELANE, IFI44, LCN2, S100A12 (S100 Calcium Binding Protein A12), CTSG (Cathepsin G), PRTN3 (Proteinase 3), OAS3 (2′-5′-Oligoadenylate Synthetase 3), IFIT3 (Interferon Induced Protein With Tetratricopeptide Repeats 3), AZU1 (Azurocidin 1) and GBP1 (Guanylate Binding Protein 1) with top 10 highest average semantic similarities were screened (Figure 6A).

We then considered the intersection of the two results above, specifically ELANE, IFI44 and LCN2, as hub genes with higher credibility (Figure 6A). ELANE and LCN2 were involved in neutrophil activation and IFI44 was involved in the response to the virus (Figure 5). Besides, ELANE and LCN2 showed persistent increase except for the period from uncomplicated infection to sepsis and were associated with increased classical monocyte as well as decreased neutrophils and memory T cells, while IFI44 was only downregulated in septic shock and showed roughly the opposite correlations with immune cell fractions (Figures 6B, C). We noticed ELANE was the only prognostic gene therein, and further validate its correlation with worse prognosis in an external dataset (GSE185263) (Figure 6D). According to the results of GSEA, upregulated neutrophil-related processes were enriched in ELANE-high group (Figure 6E). Meanwhile, downregulated TCR signaling pathway, NK cell mediated cytotoxicity and TLR signaling pathway were enriched (Figures 6E, F).

### 3.7 Neutrophil count was associated with septic shock and prognosis

**FIGURE 7:**
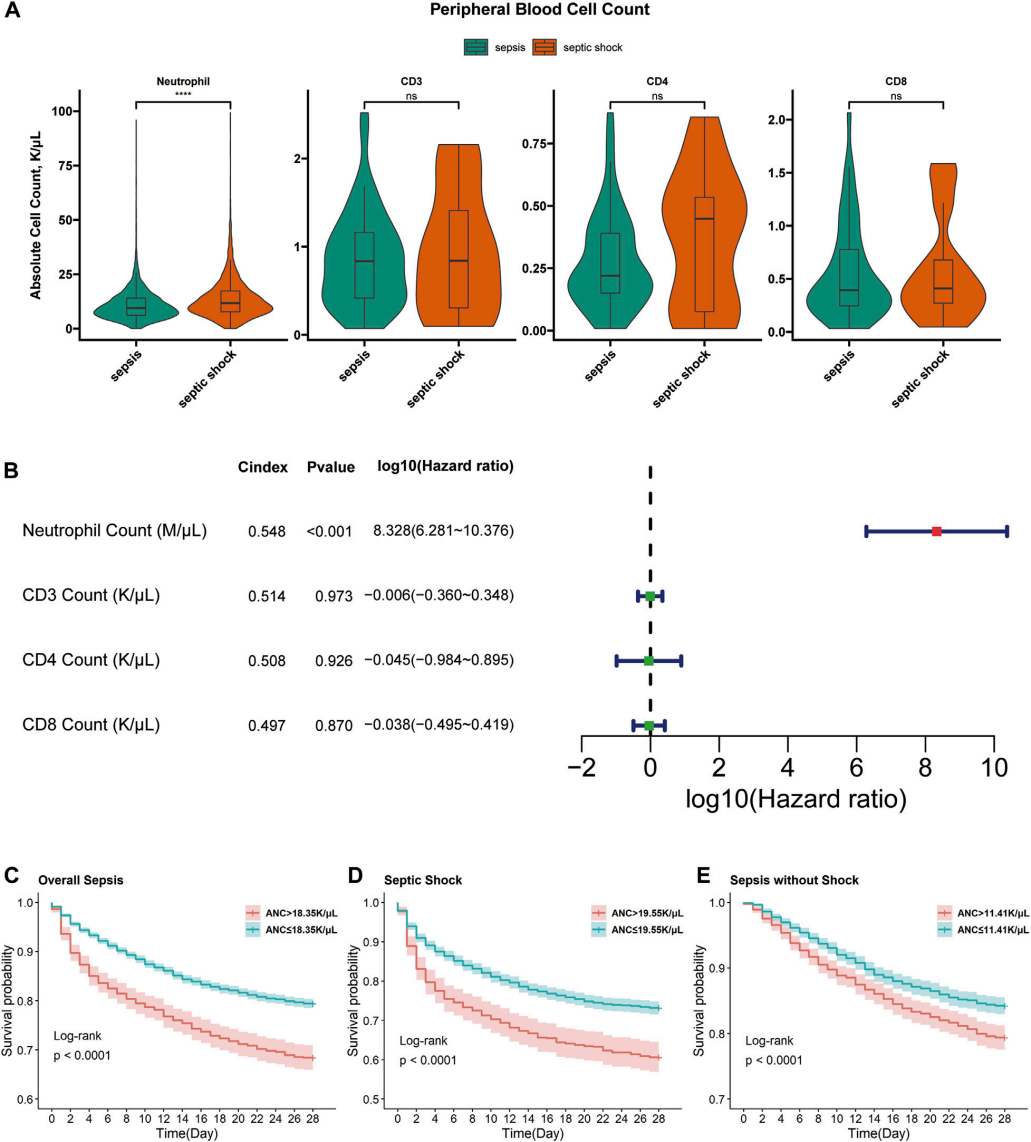
Associations of neutrophils and T cells with septic shock and prognosis of overall sepsis. **(A)** Comparisons using two-sample Wilcoxon rank sum test of neutrophil counts between sepsis (n = 4,905) and septic shock (n = 3,345) samples, CD3 count between sepsis (n = 46) and septic shock (n = 23) samples, CD4 and CD8 counts between sepsis (n = 46) and septic shock (n = 22) samples. ns, non-sense; ****, *p* < 0.0001. **(B)** The forrest plots of 4 cell counts for overall sepsis samples (n = 8,250 for neutrophil count, n = 69 for CD3 count and n = 68 for CD4 and CD8 count). Red forest plots represent risky factors and green forest plots represent protective factors. **(C)** The KM curves accessing the prognostic association of absolute neutrophil count (ANC) in overall sepsis samples. Samples with ANC>18.35 K/uL (n = 1,301) had higher 28-day cumulative mortality than samples with ANC≤18.35 K/µL (n = 6,949) (31.59% vs. 20.56%, *p* < 0.0001). **(D)** The KM curves accessing the prognostic association of ANC in septic shock samples. Samples with ANC>19.55 K/µL (n = 640) had higher 28-day cumulative mortality than samples with ANC≤19.55 K/µL (n = 2,705) (39.38% vs. 26.84%, *p* < 0.0001). **(E)** The KM curves accessing the prognostic association of ANC in non-shock sepsis samples. Samples with ANC>11.41 K/µL (n = 1827) had higher 28-day cumulative mortality than samples with ANC≤11.41 K/µL (n = 3,078) (20.63% vs 15.76%, *p* < 0.0001).

A significant difference of neutrophil counts between septic shock and sepsis patients was validated (Figure 7A). Neutrophil counts were higher in septic shock than sepsis (13.70 vs. 10.80, *p* < 0.001). Further univariate Cox analysis demonstrated the prognostic significance of neutrophil count (Figure 7B). Moreover, KM curves showed the prognostic associations of neutrophil counts as a risky factor were not only significant for overall sepsis, but also significant for septic shock and non-shock sepsis (Figures 7C–E). However, we did not obtain any statistically different distribution or prognostic association of CD3 count, CD4 count and CD8 count (Figures 7A, B), which could be explained by the complicated mechanism of T cell activation or might be affected by the smaller sample sizes.

## 4 Discussion

Sepsis causes life-threatening organ dysfunction (Singer et al., 2016), which places a great burden on human society (Fleischmann et al., 2016). Septic shock, as a subtype of sepsis, has a much higher mortality approaching 40%–60% than 10% of sepsis alone (Singer et al., 2016; Cecconi et al., 2018; Napolitano, 2018). It remains a big challenge to improve early and effective detection and management as well as the understanding of the mechanisms of septic shock. As Sepsis-3 revised in 2016 prompted new interests in sepsis immunobiology (Bermejo-Martin et al., 2016; Singer et al., 2016), further exploration of related molecular changes and underlying mechanisms could be helpful for better understanding and targeted therapy of septic shock. Most of the previous peripheral blood studies of sepsis were focused on identifying potential biomarkers, signatures, or endotypes (Scicluna et al., 2017; Baghela et al., 2022). However, since septic shock is characterized by circulatory and cellular metabolism abnormalities (Singer et al., 2016), peripheral leukocytes could be responsible for the development of septic shock.

Therefore, in the present study, we analyzed differences at the transcriptome level of peripheral blood between septic shock and sepsis. We found a significantly upregulated neutrophil activation and a dysregulated T cell activation at septic shock. The former was more likely to be associated with hyperinflammation and the latter could partially be related to suppressed interaction process of TCR and HLA-II. Interestingly, it could be inferred from the trends of module eigengenes among the step course from healthy to septic shock that neutrophils were activated when initially stimulated by infection, partially suppressed when sepsis (organ dysfunction) develops and finally abnormally reactivated involving the NETs formation under the situation of septic shock, while T cell activation showed more complicated changes involving modules with different trends. These could explain the phenotypic change patterns of these 2 cells at the different status from healthy to septic shock. A retrospective cross-sectional study has identified the neutrophil lymphocyte ratio (NLR) as a predictor of mortality and antibiotic responsiveness in ICU patients with septic shock and sepsis (Sari et al., 2019), suggesting both disorders as an important part of the mechanism of septic shock.

Neutrophils has been considered to play important and central roles during the early development of septic shock (Stiel et al., 2018). Neutrophils are known to acquire an immunosuppressive action during septic shock and participate in the generation of DIC where NETs exceed the regulatory and take an essential part (McDonald et al., 2017; Stiel et al., 2018). On the other hand, neutrophil activation was also significantly enriched in upregulated DEGs of septic shock compared with non-septic shock (Martinez-Paz et al., 2021), indicating the specific participation of excessive neutrophil activation in septic shock. We further demonstrated its most correlation with septic shock at the transcriptome level, and we found most of the prognostic SSAGs were concentrated in the neutrophil-related modules and cluster, which further revealed the major association of neutrophil with the high mortality of septic shock. Moreover, we validated the association of neutrophil count with the development and prognosis of septic shock based on the clinical data, indicating the great potential of neutrophil to help recognizing high-risk patients, and the prospect as an important line of the further target therapy research.

Septic shock is a time-dependent disease (Peltan et al., 2017). Early recognition of septic shock and effective targeted therapy in time could make sense to the practice of precision medicine thus is helpful to decrease the mortality of septic shock patients. Therefore, we identified ELANE, IFI44 and LCN2 as hub genes with the most connectivity, which have the potential to be more sensitive biomarkers for the detection of septic shock. ELANE and LCN2 were enriched in neutrophil related processes, while IFI44 was involved in adaptive-immune-response-related PPI cluster. As one of the prognostic genes, we noticed that ELANE was included in M4, enriched in the NET formation pathway and significantly correlated with neutrophil fractions, which could be the core part responsible for the high mortality. It encodes neutrophil elastase (NE), which is a serine protease and plays a critical role in innate host defense such as microbial killing (Horwitz et al., 1999; Voynow and Shinbashi, 2021). Under pathological conditions, NE, as one of the components of NETs, is released out of control during septic shock and has been proven to participate in multiple important mechanisms, such as chromatin decondensation and fibrinogenesis promotion (Massberg et al., 2010; Papayannopoulos et al., 2010). Besides, ELANE has been discussed to be involved specifically in the pyroptosis of neutrophil through mediating the cleavage and activation of GSDMD (Kambara et al., 2018), consistent with our findings about the correlation of upregulated ELANE with increased neutrophil activation and decreased neutrophil fraction. It has been proven that inhibition of NE synthesis can inhibit NET formation, reduce lipopolysaccharide (LPS)-induced acute lung injury in rats (Hagiwara et al., 2008; Okeke et al., 2020) and can significantly improve the survival rate of post-CLP septic rats (Kitamura et al., 1994), suggesting the great translational potential of ELANE as an important therapeutic target of septic shock. Moreover, previous transcriptomic studies have been published about identifying ELANE as an important signature related to the severity (SOFA score) (Baghela et al., 2022), and prognosis (Ding et al., 2022; Zhang et al., 2022), of sepsis patients. We further demonstrated the correlation of ELANE with septic shock and its vital participation in the core mechanism of septic shock. As for the other two, LCN2 encodes a secreted protein called neutrophil gelatinase-associated lipocalin (NGAL). It can be stimulated by Toll-like receptors and is pivotal in the innate immune response to bacterial infection through binding bacterial siderophores (Flo et al., 2004). LCN2 has been proven to differentially expressed between septic shock and sepsis in surgical patients (Martin-Fernandez et al., 2020), and has been reported as a potential biomarker of septic-shock-associated acute kidney injury (Tang et al., 2021). IFI44 is an interferon-alpha inducible protein associated with infection by several viruses (Power et al., 2015). The downregulation of IFI44 could in a way represent the suppressed adaptive immune response in septic shock. The specific roles of IFI44 in septic shock have not been defined yet. In summary, ELANE and LCN2 were enriched in neutrophil activation and correlated with infection and septic shock, especially ELANE as the only prognostic gene could participate through NETs formation and pyroptosis pathways. IFI44 was associated with adaptive immune response and specifically downregulated in septic shock. All three hub genes did not show any significant change between sepsis and uncomplicated infection.

Our study is the first in our knowledge to focus on the mechanisms about the contribution of peripheral leukocyte to the development of septic shock under the new Sepsis-3 definition since 2016. The roles of neutrophil activation and NETs in septic shock have been reported in previous studies. We further demonstrated their most correlation with septic shock combining the WGCNA and PPI network analysis. We highlighted septic shock as a subset of sepsis with much higher mortality showing different expression profiles in peripheral blood. The distinct distribution bias that most of the septic-shock-associated prognostic genes were concentrated in neutrophil-related modules and PPI cluster may be worthy of note. However, there are still some limitations of the study. The relatively small sample size of GSE154918 could partially limit the universal implication of our findings, however the consistency between the results of co-expression network and PPI network analysis could up to a point improve the credibility and represent an important endotype of septic shock. More importantly, the specific phenotypic changes of neutrophil and T cells, for example, whether ELANE was actually or only upregulated in neutrophil, and whether or how ELANE-mediated pyroptosis of neutrophil take part in were still unclear. The underlying cellular heterogenicity might partially reduce the credibility of our findings and the further well-designed research with directed focus is still needed.

Overall, septic shock showed an excessive neutrophil activation and a dysregulated T cell activation, of which the former was associated with hyperinflammation and the latter could partially be related to suppressed interaction process of TCR and HLA-II. Neutrophil activation may play a core role during septic shock. ELANE, LCN2 and IFI44 were identified as hub genes during septic shock, among which ELANE as a neutrophil-related gene might have the greatest potential to be a clinical biomarker and therapeutic target. This study highlighted an important perspective about septic shock under the new definition and would help in designing further translational research to improve diagnosis and treatment.

## Data Availability

The datasets presented in this study can be found in online repositories. The names of the repository/repositories and accession number(s) can be found in the article/Supplementary Material.

## Glossary

DEG: Differentially expressed gene
SSAG: Septic-shock-associated gene
BH: Benjamini–Hochberg method
WGCNA: weighted gene co-expression network analysis
PPI: protein–protein interaction network
CMR: cumulative mortality rate
adj.P: adjusted *p*-value
BH: Benjamini–Hochberg method
GSEA: gene set enrichment analysis
GEO: gene expression omnibus
logFC: log2 fold change
BP: biological process
TCR: T cell receptor
HLA: Human leukocyte antigen
HLA-DPA1: Major Histocompatibility Complex, Class II, DP Alpha 1
HLA-DQA1: Major Histocompatibility Complex, Class II, DQ Alpha 1)
HLA-DRB1: Major Histocompatibility Complex, Class II, DR Beta 1
HLA-DMB1: Major Histocompatibility Complex, Class II, DM Beta 1
HLA-DPB1: Major Histocompatibility Complex, Class II, DP Beta
ELANE: Elastase, Neutrophil Expressed
IFI44: Interferon Induced Protein 44
LCN2: Lipocalin 2
DEFA4: Defensin Alpha 4
IFIT1: Interferon Induced Protein With Tetratricopeptide Repeats 1
MMP8: Matrix Metallopeptidase 8
MPO: Myeloperoxidase
MX1: MX Dynamin Like GTPase 1
RSAD2: Radical S-Adenosyl Methionine Domain Containing 2
S100A12: S100 Calcium Binding Protein A12
CTSG: Cathepsin G
PRTN3: Proteinase 3
OAS3: 2′-5′-Oligoadenylate Synthetase 3
IFIT3: Interferon Induced Protein With Tetratricopeptide Repeats 3
AZU1: Azurocidin 1
GBP1: Guanylate Binding Protein 1
DIC: disseminated intravascular coagulation
NET: neutrophil extracellular trap
NE: neutrophil elastase.
