# Supplementary material for: Co-expression and interaction network analysis reveals dysregulated neutrophil and T-cell activation as the core mechanism associated with septic shock
Source: Front Genet. 2023 Feb 23;14:1132361. doi: 10.3389/fgene.2023.1132361 (PMC9997678; doi:10.3389/fgene.2023.1132361)
Supplement: Supplementary file 1 [file Presentation1.pdf]

Supplementary Material

TableS1. Demographic and clinical characteristics of included ICU stays

	**Neutrophil Count n=8250**			**CD3 Count, n=69**			**CD4 and CD8 Count, n=68**		
	**Sepsis n=4905**	**Septic shock n=3345**	**P value[b]**	**Sepsis n=46**	**Septic Shock n=23**	**P value[b]**	**Sepsis n=46**	**Septic Shock n=22**	**P value[b]**
Age, median [Q1, Q3], years	67.37 [56.41, 78.08]	68.41 [58.13, 77.51]	0.072	55.53 [50.64, 63.85]	53.29 [42.81, 62.25]	0.207	55.53 [50.64, 63.85]	52.58 [41.97, 61.77]	0.145
Male, n (%)	2806 (57.21%)	2033 (60.78%)	0.001	37 (80.43%)	16 (69.57%)	0.313	37 (80.43%)	15 (68.18%)	0.265
SOFA score[a], median [Q1, Q3]	3 [2, 4]	4 [3, 6]	<0.001	3 [2, 4]	4 [3, 6]	0.033	3 [2, 4]	4 [3, 5.75]	0.052
Blood gas lactate[a], median [Q1, Q3], mmol/L	1.8 [1.4, 2.8]	3.8 [2.7, 6.1]	<0.001	2 [1.4, 3.825]	4 [3.35, 9.05]	<0.001	2 [1.4, 3.825]	3.9 [3.325, 8.425]	<0.001
Mortality 28 days, n (%)	862 (17.57%)	978 (29.24%)	<0.001	9 (19.57%)	9 (39.13%)	0.081	9 (19.57%)	9 (40.91%)	0.062

a. The first-day maximum values were extracted for these parameters.
b. P values were calculated using Wilcoxon rank sum test for variables data and Pearson's Chi-squared test for attributes data.

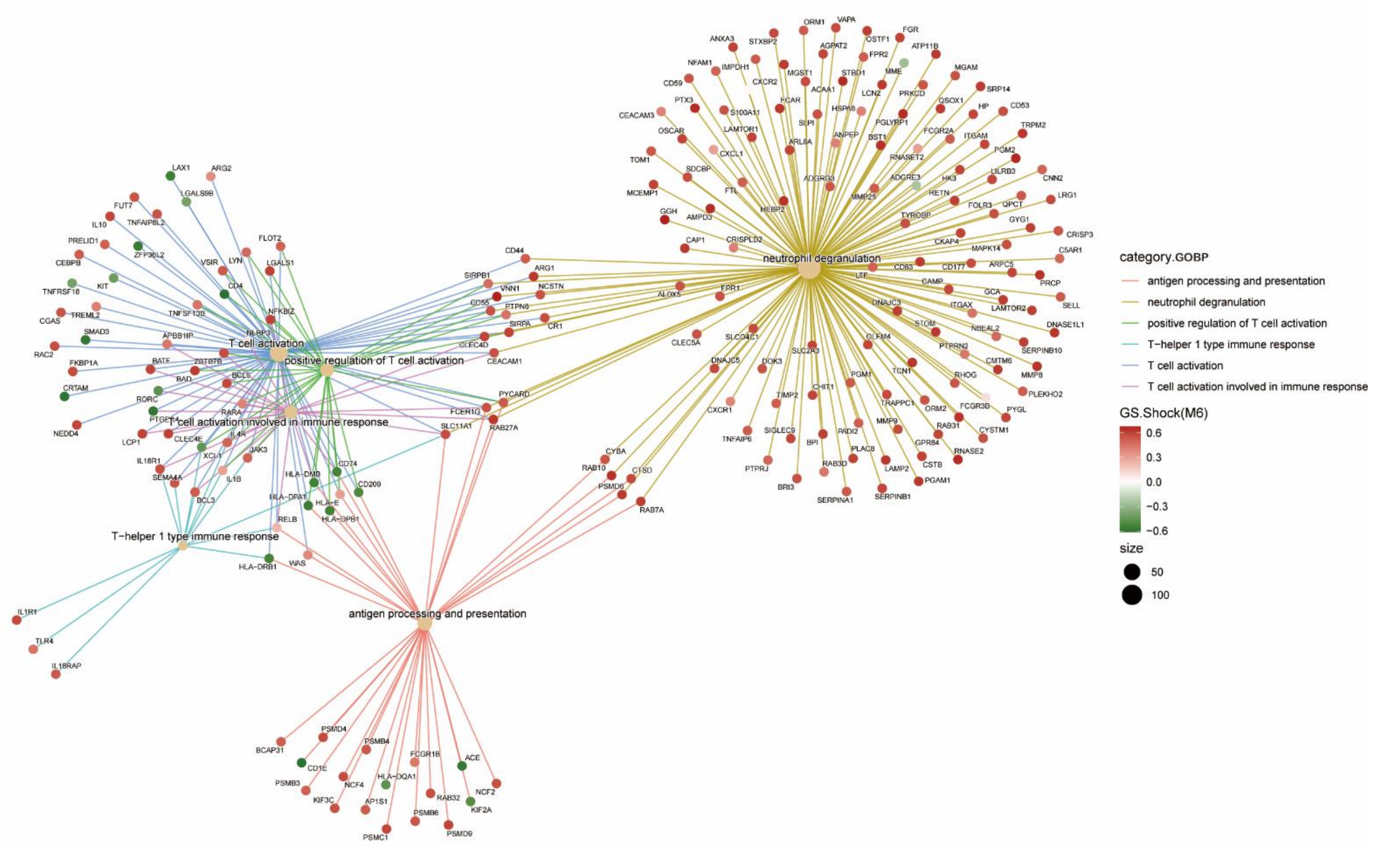

Figure S1. Network of enriched GO biological processes and involved genes of M6 module. The size of category nodes indicated the number of genes enriched in the corresponding terms, and the color of gene nodes indicated the corresponding gene significance (GS) for septic shock.

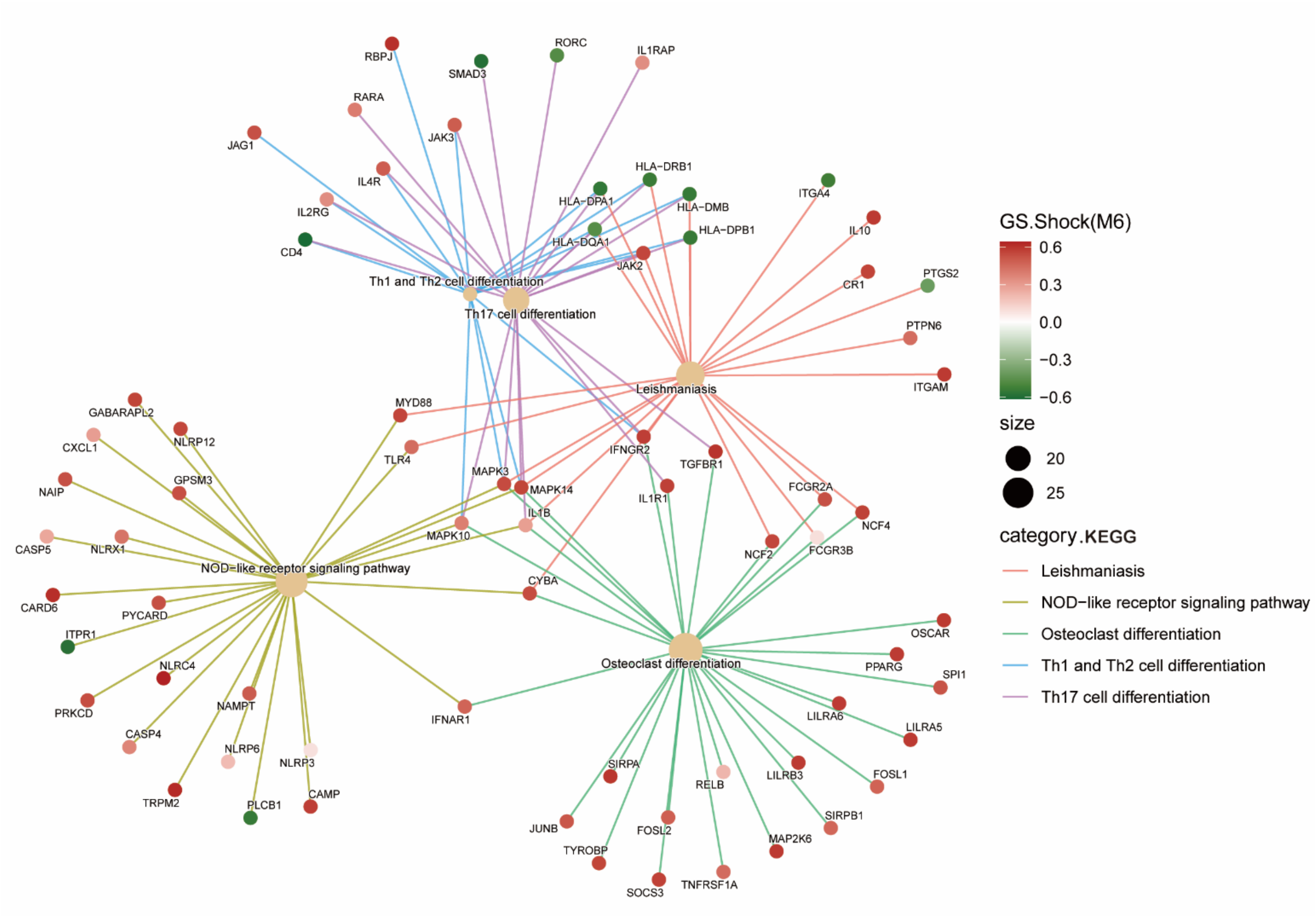

Figure S2. Network of enriched KEGG pathways and involved genes of M6 module. The size of category nodes indicated the number of genes enriched in the corresponding terms, and the color of gene nodes indicated the corresponding gene significance (GS) for septic shock.
